# *Rickettsia helvetica* infection is associated with microbiome modulation in *Ixodes ricinus* collected from humans in Serbia

**DOI:** 10.1038/s41598-022-15681-x

**Published:** 2022-07-06

**Authors:** Apolline Maitre, Alejandra Wu-Chuang, Lourdes Mateos-Hernández, Angélique Foucault-Simonin, Sara Moutailler, Jean-Christophe Paoli, Alessandra Falchi, Adrian A. Díaz-Sánchez, Pavle Banović, Dasiel Obregón, Alejandro Cabezas-Cruz

**Affiliations:** 1 ANSES, INRAE, Ecole Nationale Vétérinaire d’Alfort, UMR BIPAR, Laboratoire de Santé Animale, 94700 Maisons-Alfort, France; 2 INRAE, UR 0045 Laboratoire de Recherches Sur Le Développement de L’Elevage (SELMET-LRDE), 20250 Corte, France; 3 EA 7310, Laboratoire de Virologie, Université de Corse, Corte, France; 4 Department of Biology, University of Saskatchewan, 112 Science Place, Saskatoon, SK S7N 5E2 Canada; 5 Ambulance for Lyme Borreliosis and Other Tick-Borne Diseases, Pasteur Institute Novi Sad, 21000 Novi Sad, Serbia; 6 Department of Microbiology With Parasitology and Immunology, Faculty of Medicine, University of Novi Sad, 21000 Novi Sad, Serbia; 7 School of Environmental Sciences, University of Guelph, Guelph, ON Canada

**Keywords:** Bacteria, Microbial communities, Pathogens

## Abstract

*Rickettsia helvetica* is an emerging pathogen of the Spotted Fever Group Rickettsia (SFGR) causing spotted fever diseases in various European countries. This tick-borne pathogen replicates in tick tissues such as the midgut and salivary gland, but its potential interactions with the vector microbiota is poorly characterized. The vector microbiome plays a pivotal role in tick-pathogen interactions, and some microbiota members facilitate or impede tick-borne pathogen infection. Manipulations of the tick microbiome have led to reduction in pathogen colonization in the tick vector. However, translating these findings into disease control applications requires a thorough characterization of vector microbiota response to different pathogens. In this study, we analyzed and compared the microbiota of *Ixodes ricinus* ticks attached on humans and collected in Serbia. Ticks were either infected with *R. helvetica*, or uninfected with major tick-borne pathogens (referred hereafter as ‘pathogen-free’). We used microbial co-occurrence network analysis to determine keystone taxa of each set of samples, and to study the interaction patterns of the microbial communities in response to pathogen infection. The inferred functional profiles of the tick microbiome in *R. helvetica*-positive and pathogen-free samples were also compared. Our results show that *R. helvetica* infection reduces significantly the diversity of the microbiota and the connectivity of the co-occurrence network. In addition, using co-occurrence network we identified bacterial taxa (i.e., Enterobacteriaceae, Comamonadaceae, and *Bacillus*) that were negatively associated with ‘*Rickettsia*’ in *R. helvetica*-infected ticks, suggesting competition between *R. helvetica* and some members of the tick microbiota. The reconstruction of microbial metabolic pathways shows that the presence of *R. helvetica* might have a major impact on the metabolic functions of the tick microbiome. These results can inform novel interventions for the prevention of *R. helvetica*, or other SFGR infections in humans.

## Introduction

Ticks are hematophagous ectoparasites of vertebrate hosts and transmit the greatest variety of pathogens including bacteria, viruses and protozoa^1,2^. Among the approximately 900 known tick species, at least 10% have medical or veterinary importance^3^. *Ixodes ricinus* is the most widespread hard tick species in Europe^4^, where it transmits the spirochete *Borrelia burgdorferi*^5^, intracellular bacteria of the genera *Rickettsia*, *Anaplasma* and *Ehrlichia*^6^, viruses such as tick-borne encephalitis virus (TBEV), and parasites such as *Babesia* spp.^7^.

The genus *Rickettsia* includes 32 species of obligate intracellular bacteria, including 18 that are human pathogens^8^, of which 8 are transmitted by ticks in Europe^9^. Bacteria in the genus *Rickettsia* are separated in four groups, two of which, the typhus group *Rickettsia* and the spotted fever group *Rickettsia* (SFGR), contain pathogenic bacteria. *Rickettsia helvetica* belongs to the SFGR with its main vector in Europe being *I. ricinus* which can also be a natural reservoir^10^. *Rickettsia helvetica* has also been found in *Ixodes hexagonus* in Germany, in *Ixodes arboricola* in Czech Republic and in *Dermacentor reticulatus* in Croatia^10^, but their role as vectors for this bacterium is not unequivocally recognized. *Rickettsia helvetica* can be acquired through an infected bloodmeal by larvae and are transmitted both transstadially and transovarially^11^. This bacterium is an emerging human pathogen suspected to cause fever, headache, arthralgia and myalgia^12^. In addition to being found in Europe^9^, *R. helvetica* is present in northern Africa and Asia^10,12^. *Rickettsia helvetica* has been detected in 7.7% to 54% of tested *I. ricinus* ticks in Serbia^4,13^.

In addition to pathogens, tick tissues (e.g., guts and salivary glands) are colonized by commensal and mutualistic bacteria that, along with archaea, fungi and viruses, compose the tick microbiota^14,15^. Bacterial microbiota composition varies according to tick species^16,17^, tick organ, sex, life stage^16,18–22^, feeding status^23^, and the host species used for feeding^24,25^. Environmental factors are also shown to influence tick microbiota composition^16,17,19–21^. Despite high taxonomic variability, some bacterial taxa including *Arsenophonus*, *Candidatus* Midichloria, *Rickettsia* and *Wolbachia* among others have been frequently identified in *I. ricinus*^26^.

Pathogenic bacteria transmitted by *Ixodes* ticks are known to modulate the tick microbiota to facilitate pathogen colonization in the tick vector^27,28^. For example, *B. burgdorferi*^27^ and *Anaplasma phagocytophilum*^28^ colonization in *Ixodes scapularis* is associated with changes in the taxonomic composition of the microbiota resulting in diffuse biofilms in the tick midguts. Particularly, *B. burgdorferi* or *A. phagocytophilum* infection triggers the expression of *I. scapularis* genes in the tick midgut cells. A secreted protein with a Reeler domain (PIXR) and an antifreeze glycoprotein (IAFGP) with microbiota-modulatory properties are upregulated by *B. burgdorferi* and *A. phagocytophilum*, respectively^27,28^. This provides a molecular basis to explain tick microbiota modulation by tick-borne pathogens and indirect interactions between bacterial microbiota and the intracellular and extracellular pathogens transmitted by ticks.

**Figure 1:**
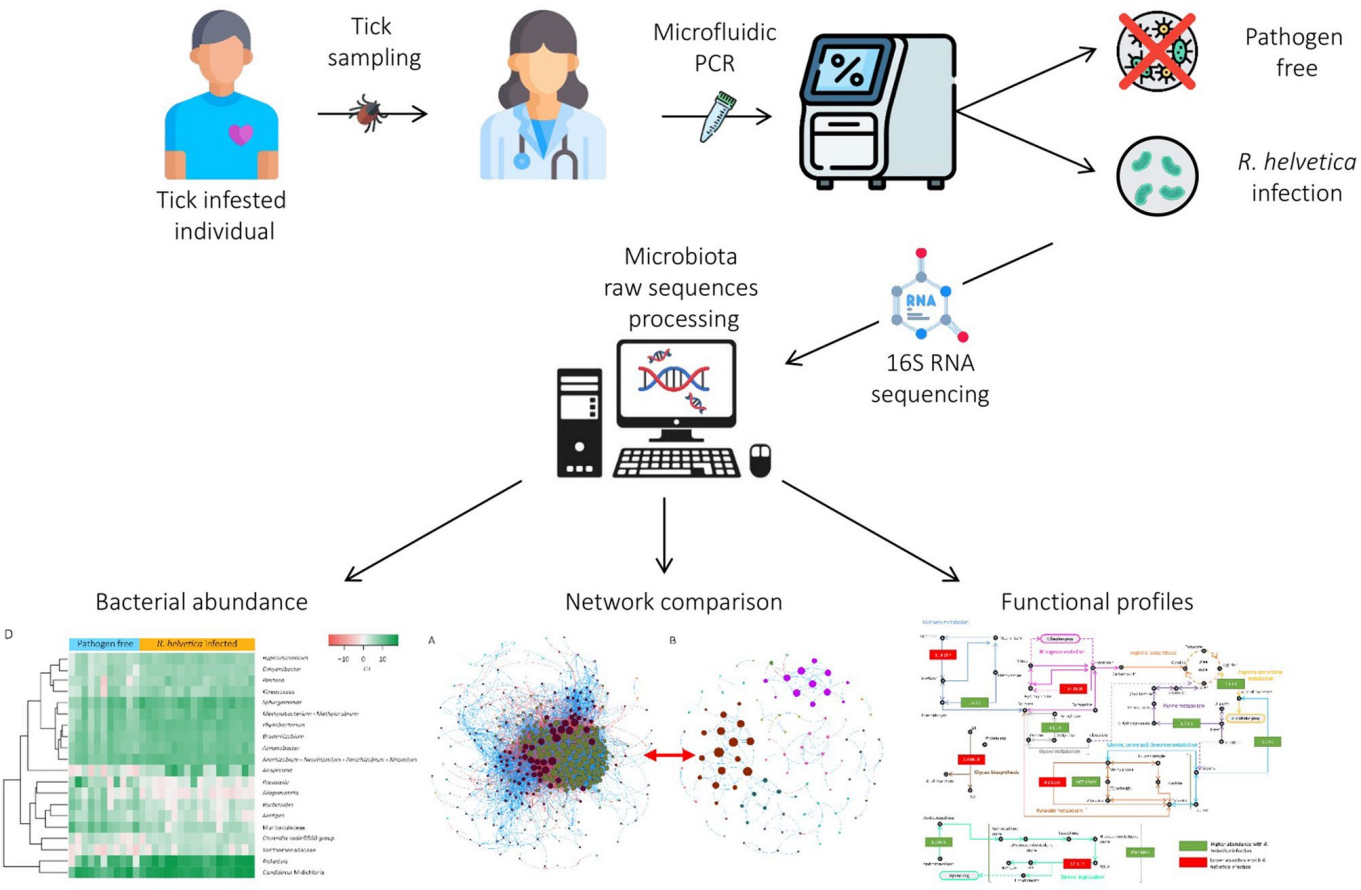
Experimental design. Ticks used in this study were collected from humans in Serbia, and the presence of tick-borne pathogens was assessed by microfluidic PCR^4^. Ticks were either infected with *R. helvetica*, or uninfected by major tick-borne pathogens (referred hereafter as ‘pathogen-free’). In this study, the *R. helvetica*-infected and pathogen-free DNA samples were used for 16S rRNA amplicon sequencing and microbiome analysis. Bacterial composition and abundance were analyzed. The alpha-, beta-diversity and beta-dispersion of the two datasets were compared, and co-occurrence networks were inferred to assess the structure of the microbial communities. Functional gene prediction in *R. helvetica*-infected and pathogen-free ticks was performed using PICRUSt2 and explored for possible impact of *R. helvetica* infection on metabolic traits of the tick microbiome.

In a recent study, Lejal et al.^26^ compared the microbiota of *I. ricinus* nymph over a period of three years (from 2014 to 2017), and documented marked shifts in tick microbiota dynamics over time. These authors also showed significant correlations between the presence of specific pathogens and the structure of the *I. ricinus* microbiota. In particular, their results suggested that *R. helvetica* drives strong interactions in the tick microbiome^26^. In this study, we analyzed and compared the microbiota of uninfected and *R. helvetica*-infected *I. ricinus* ticks collected on humans in Serbia (Fig. 1). Our results show that *R. helvetica* infection has a significant impact on the taxonomic and inferred functional profiles of tick microbiota. The findings of some taxa that correlate positively with *R. helvetica* concur with those by Lejal et al.^26^ and can inform novel interventions for the prevention of *R. helvetica* infection in humans.

## Results

### Impact of *R. helvetica* infection on bacterial diversity of *I. ricinus* microbiota

**Figure 2:**
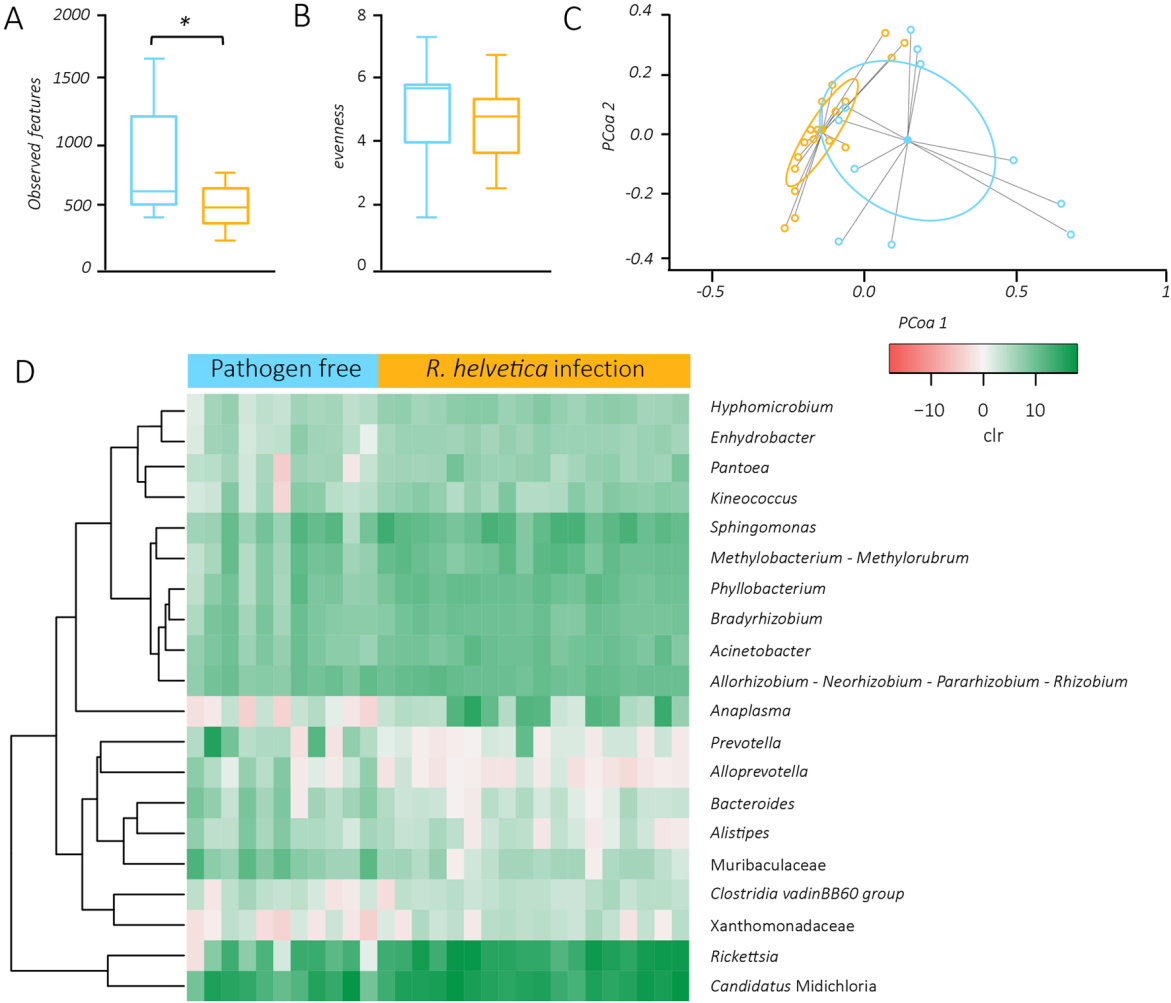
Impact of *R. helvetica* infection on tick microbial diversity and taxa bacterial abundance. (**A**) Comparison of alpha-diversity with observed features index for *R. helvetica*-infected (orange) and pathogen-free (blue) samples (*p = 0.015). (**B**) Comparison of alpha-diversity with evenness index for *R. helvetica*-infected (orange) and pathogen-free (blue) samples (*p* = 0.393). (**C**) Comparison of beta-diversity with Bray Curtis dissimilarity index for *R. helvetica*-infected (orange) and pathogen-free (blue) samples. Small circles represent samples, and ellipses represent centroid position for each group (*F* = 5.313; *p* = 0.001; stress = 0.130). Beta dispersion of the two sets of samples. Small circles represent samples, and ellipses represent dispersion of samples. This test use Principal Coordinate Analysis (PCoA), it is used to explore and to visualize variability in a microbial community. ANOVA test was performed and showed that beta dispersion of the two sets of samples is significantly different (*p* = 4.775e−05). (**D**) Dendrogram heatmap resulting from the heatmap.2 function implemented on R studio^73^. The taxa were clustered based on relative abundance (calculated as clr transformed values). Each column represents the clr values for bacterial taxa per sample and per group. Each line represents bacterial taxa with significant changes between the two datasets. Color represent the clr value.

Analysis of bacterial diversity using “observed features” (number of different amplicon sequence variants (ASV) observed in the sample) revealed that the microbiota of *I. ricinus* ticks infected with *R. helvetica* showed a significant decrease of microbial richness compared to that of pathogen-free ticks (Kruskal–Wallis, *p* < 0.05, Fig. 2A). The evenness index was not significantly different between the groups (Fig. 2B). Bray–Curtis index demonstrated that the two sets of samples have different community composition (PERMANOVA, *p* ≤ 0.001, Fig. 2C). Further, pathogen-free samples were more different to each other than the *R. helvetica*-positive samples (Beta dispersion tested with ANOVA test, *p* < 0.001, Fig. 2C). The *Rickettsia* taxon was present in 100% of the *R. helvetica*-positive samples, in agreement with the positive microfluidic results of *R. helvetica* in our samples. *Rickettsia* was also present in 73% of the pathogen-free samples (8 out of 11), but the presence of the pathogens *R. helvetica, R. conorii, R. slovaca, R. massiliae, R. aeschlimannii and R. felis* was ruled out based on microfluidic PCR analysis. This taxon was also more abundant in *R. helvetica-*infected samples (Ave 36.25 ± 22.40 centered log ratio (clr)) than in pathogen-free samples (Ave 3.91 ± 4.13 clr). Differential relative abundance (expressed as clr) analysis showed that 20 taxa changed significantly between the two datasets. Some taxa were significantly more abundant in *R. helvetica*-positive samples (i.e., *Hyphomicrobium*, *Enhydrobacter*, *Pantoea, Kineococcus*, *Sphingomonas*, *Methylobacterium*—*Methylorubrum*, *Phyllobacterium*, *Bradyrhizobium*, *Acinetobacter*, *Allorhizobium*—*Neorhizobium*—*Pararhizobium*—*Rhizobium*, *Ca.* Midichloria, *Anaplasma*, *Clostridia* vadinBB60 group, Xanthomonadaceae and *Rickettsia*), while others were predominant in pathogen-free samples (i.e., *Prevotella, Alloprevotella, Bacteroides, Alistipes* and Muribaculaceae) (Fig. 2D).

### Impact of *R. helvetica* infection on the structure of microbial communities of *I. ricinus* microbiota

**Figure 3:**
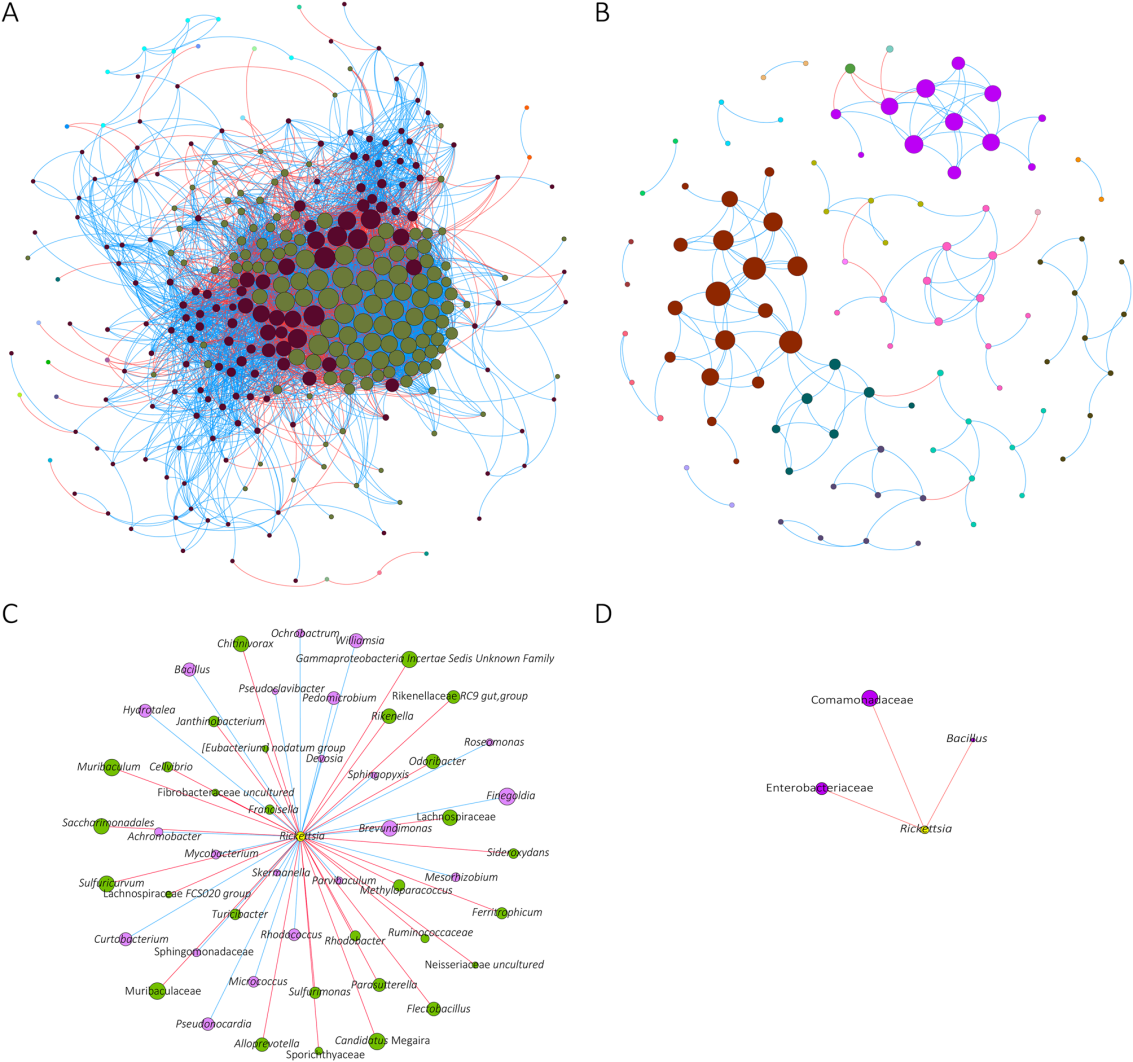
Global and local co-occurrence networks. Co-occurrence networks of (**A**) pathogen-free and (**B**) *R. helvetica*-positive samples. The direct neighbors of *Rickettsia* were identified in the bacterial co-occurrence networks of (**C**) pathogen-free and (**D**) *R. helvetica*-positive samples and represented as sub-networks. Nodes correspond to taxa (family or genera level), and connecting edges indicate significant connection between them (*p* < 0.01). Only nodes with at least one significant correlation are represented. Node colors are based on modularity class metric and equal color means modules of co-occurring taxa. The size of the nodes is proportional to the eigenvector centrality of each taxon. The colors in the edges represent strong positive (blue) or negative (red) correlations (SparCC > 0.75 or < − 0.75).

We asked whether in addition to changes in bacterial composition, richness, and relative abundance, *R. helvetica* infection has also an impact on the *I. ricinus* microbiota structure. To address this question, the microbial co-occurrence patterns were quantified using co-occurrence networks. Visually, the pathogen-free network has two large highly-connected modules composed of nodes with strong and multiple positive (blue) interactions between them. Marginal taxa with poor connectivity with the main modules were also observed in the network (Fig. 3A). The *R. helvetica*-positive network is scarcer; taxa were less connected and no large modules with strong interactions were observed (Fig. 3B). The pathogen-free network is almost 40 times more connected than the *R. helvetica*-positive one with 6112 edges for the pathogen-free ticks and 164 edges for the infected ticks (Table 1). The reduction in connectivity between taxa suggests that the decrease in bacterial abundance and diversity associated with the presence of *R. helvetica* have a significant impact on the structure of the bacteria communities making up the microbiota of *I. ricinus*. Jaccard index was used to test for similarities (Jacc = 0, lowest similarity and Jacc = 1, highest similarity) in selected local network centrality measures of the two networks. All these measures were found to be lower than expected by random for the comparison between pathogen-free and *R. helvetica*-positive networks (Table 2). The Adjusted Rand index (ARI) similarity index (see “Materials and methods”) clustering was 0.18 (*p* = 0), which confirms the low degree of similarity between the networks. These results confirmed the significant differences in topology between these networks. Topological differences, together with dissimilarity in local network centrality measures, indicate a major shift in the community structure induced by the presence of *R. helvetica* in the ticks.

**Table 1:** Topological features of the taxonomic networks with and without *R. helvetica* presence.

Topological features	*Ixodes ricinus*	
	Pathogen-free	*R. helvetica* infection
Nodes	713	501
Edges	6112	164
Positives	4056 (66%)	155 (95%)
Negatives	2056 (34%)	9 (5%)
Modularity	1.368	0.836
Network diameter	9	10
Average degree	17.144	3.28
Weighted degree	4.66	2.373
Clustering coefficient	0.644	0.525

**Table 2:** Jaccard index for pathogen-free and *R. helvetica* infection networks.

	Jaccard index	*P* (≤ Jacc)	*P* (≥ Jacc)
Degree	0.210	2^e−05^***	0.999
Betweenness centrality	0.173	2^e−06^***	0.999
Closeness centrality	0.210	2^e−05^***	0.999
Eigenvector centrality	0.147	0 ***	1
Hub taxa	0.147	0 ***	1

The impact of *R. helvetica* infection on the local connectivity of *Rickettsia* was visualized using sub-networks representing the direct neighbors around this taxon in pathogen-free (Fig. 3C), and *R. helvetica*-positive (Fig. 3D) samples. Visual inspection showed differences in the connectivity of *Rickettsia*. This taxon was positively and negatively connected with 21 and 29 taxa, respectively in the pathogen-free network (Fig. 3C), whereas in the *R. helvetica*-positive network, *Rickettsia* was only connected to three taxa (i.e., Enterobacteriaceae, Comamonadaceae and *Bacillus*) (Fig. 3D). The correlation between *Rickettsia* and Enterobacteriaceae and Comamonadaceae was negative. Notably, *Bacillus* was identified in both networks, and it was positively and negatively correlated with *Rickettsia* in the pathogen-free and *R. helvetica*-positive sub-networks, respectively. The results suggest that *R. helvetica* reduces the microbe-microbe interactions in the bacterial microbiota of *I. ricinus*.

### Impact of *R. helvetica* infection on the hierarchical organization of *I. ricinus* microbiota

**Figure 4:**
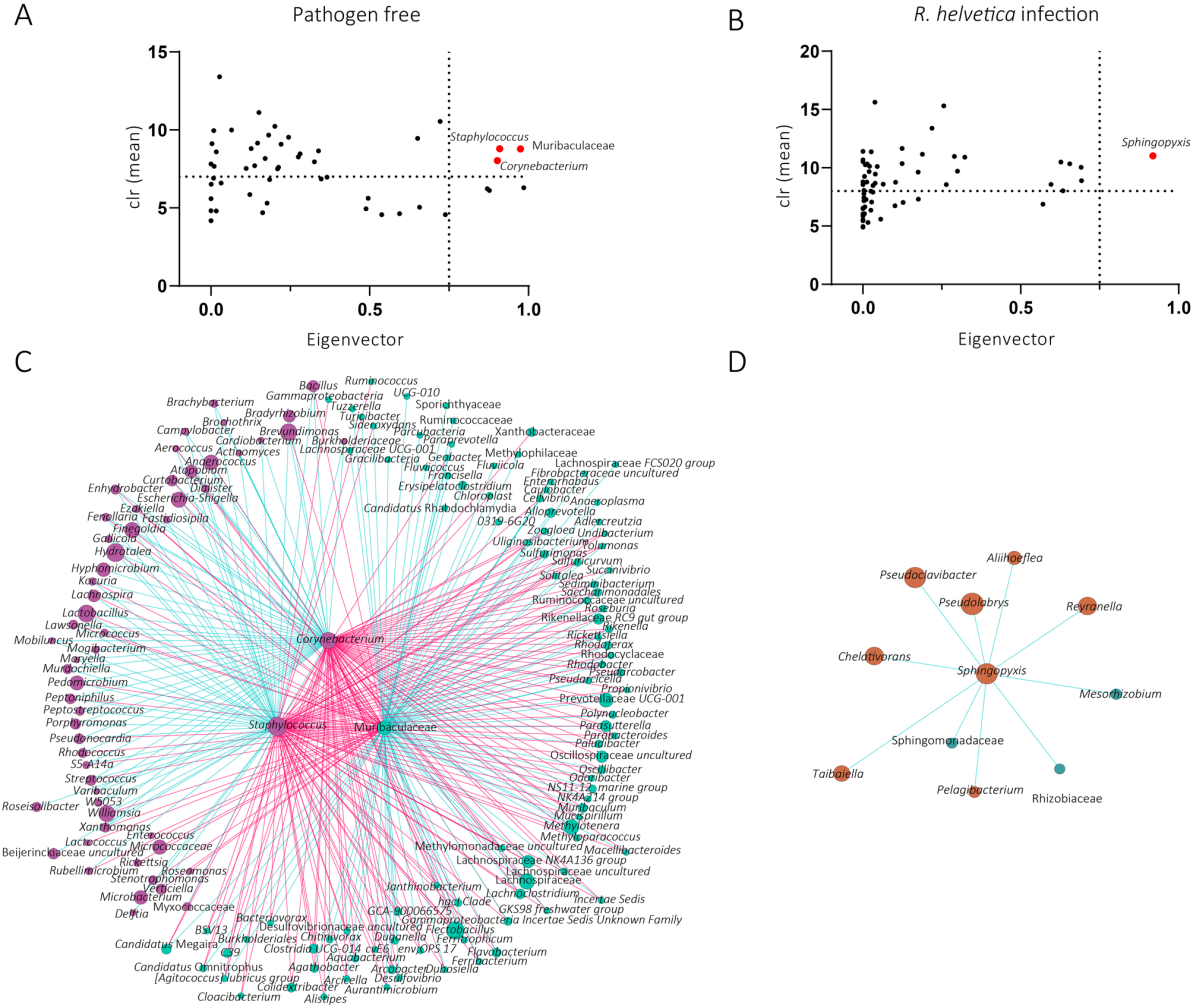
Keystone taxa in microbial communities and co-occurrence networks of the local connectivity identified in *R. helvetica*-positive and pathogen-free samples. Scatter plot of (**A**) pathogen free and (**B**) *R. helvetica*-infected datasets, showing the mean relative abundance (calculated as clr transformed values), and the eigenvector centrality of ubiquitous (present in all samples) bacterial taxa. The vertical dotted line represents the eigenvector centrality cutoff value set at 0.75, and the horizontal dotted line represents the average clr value of all taxa. Taxa were considered keystone when they were ubiquitous and with clr and eigenvector centrality values above these thresholds. Black dot represents ubiquitous taxa below the thresholds. Red dots represent keystone taxa. The taxonomic identity of the keystone taxa is presented for each group. (**C**) The direct neighbors of the keystone taxa *Staphylococcus*, *Corynebacterium* and Muribaculaceae (in the center of the circle) and (**D**) *Sphingopyxis* were identified in the co-occurrence networks from pathogen-free and *R. helvetica-*positive samples, respectively. Positive and negative interactions between co-occurring bacteria are represented by the blue and red edges, respectively.

We then asked whether the presence of *R. helvetica* in the microbial community changed the hierarchical organization of *I. ricinus* microbiota. Keystone species plays an important role in the stability of the microbial ecosystem. Keystone taxa were identified based on their ubiquitousness, high relative abundance, and high eigenvector centrality (> 0.75) in the networks. Three taxa met these criteria for the pathogen-free microbiota and one for the *R. helvetica*-positive microbiota. The pathogen-free keystone taxa were the genera *Staphylococcus* and *Corynebacterium,* and the family Muribaculaceae (Fig. 4A). The *Staphylococcus* and *Corynebacterium* taxa are positively connected, with both negatively connected to Muribaculaceae. The keystone taxon in *R. helvetica*-positive samples was *Sphingopyxis* (Fig. 4B).

To have a better idea of changes in the hierarchical organization of the microbiota, and their impact on the bacterial environment, we identified the direct neighbors of keystone taxa in pathogen-free and the *R. helvetica*-positive samples and displayed them as co-occurrence sub-networks. The pathogen-free sub-network contains 161 taxa positively or negatively connected with at least one of the keystone taxa (Fig. 4C). Specifically, positive and negative associations were found for *Corynebacterium* (45 positive and 74 negative) *Staphylococcus* (46 positive and 79 negative) and Muribaculaceae (91 positive and 35 negative). The *R. helvetica*-positive sub-network contains 10 taxa (i.e., *Pseudoclavibacter*, *Chelativorans*, *Taibaiella*, Sphingomonadaceae, *Pelagibacterium*, *Mesorhizobium*, *Reyranella*, *Aliihoeflea*, *Pseudolabrys* and Rhizobiaceae); all positively connected with *Sphingophyxis* (Fig. 4D). The two sub-networks did not share any taxa. The taxon *Rickettsia* was only identified in the pathogen-free sub-network, and it was negatively correlated with Muribaculaceae family. These results suggest that the infection of *R. helvetica* in *I. ricinus* ticks induce changes in the hierarchical organization of *I. ricinus* microbiota.

### Impact of *R. helvetica* infection on the inferred functional profiles of *I. ricinus* microbiota

**Figure 5:**
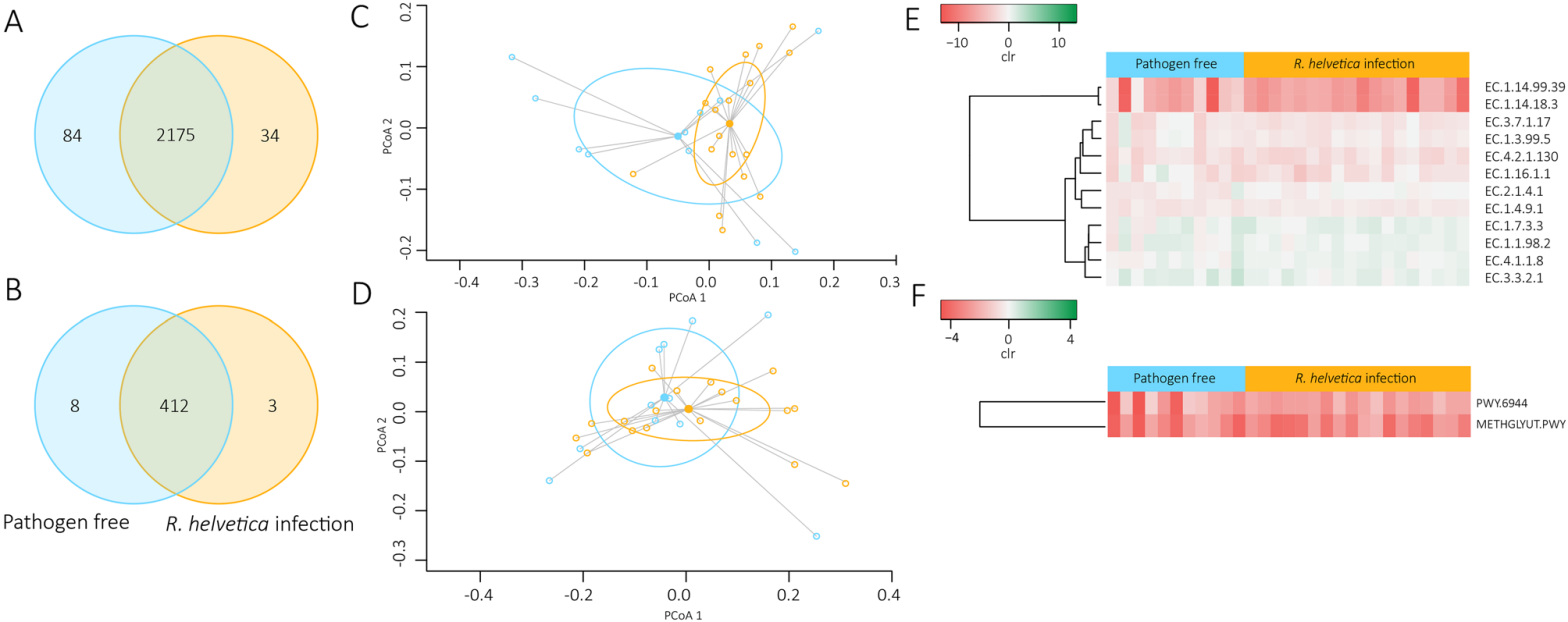
Comparison of unique and shared enzymes and pathways in the different groups and significant changes in relative abundance. A Venn diagram displaying the comparison of (**A**) enzymes and (**B**) pathways composition between the *R. helvetica-*positive and pathogen-free *I. ricinus* ticks. The blue and red circles represent the enzymes or pathways in pathogen-free or *R. helvetica-*positive samples, respectively. Numerals represent the number of enzymes or pathways found in each dataset and those shared by the two groups. (**C**) Comparison of beta-diversity with Bray Curtis dissimilarity index for enzymes of *R. helvetica* and pathogen-free samples. Small circles represent samples, and ellipses represent centroid position for each group (bray–curtis index, PERMANOVA, *p* > 0.05). Beta dispersion of the two sets of samples. ANOVA test was performed and showed that beta dispersion of the two sets of samples is significantly different (*p* = 0.008). (**D**) Comparison of beta-diversity with Bray Curtis dissimilarity index for pathways of *R. helvetica* and pathogen-free samples (bray–curtis index, PERMANOVA, *p* > 0.05). Beta dispersion of the two sets of samples. ANOVA test was performed and showed that beta dispersion of the two sets of samples is not significantly different (ANOVA, *p* = 0.08). (**E**) Heatmap of the significantly different enzymes abundance with and without *R. helvetica* presence. (**F**) Heatmap of the significantly different pathways abundance with and without *R. helvetica* presence.

**Figure 6:**
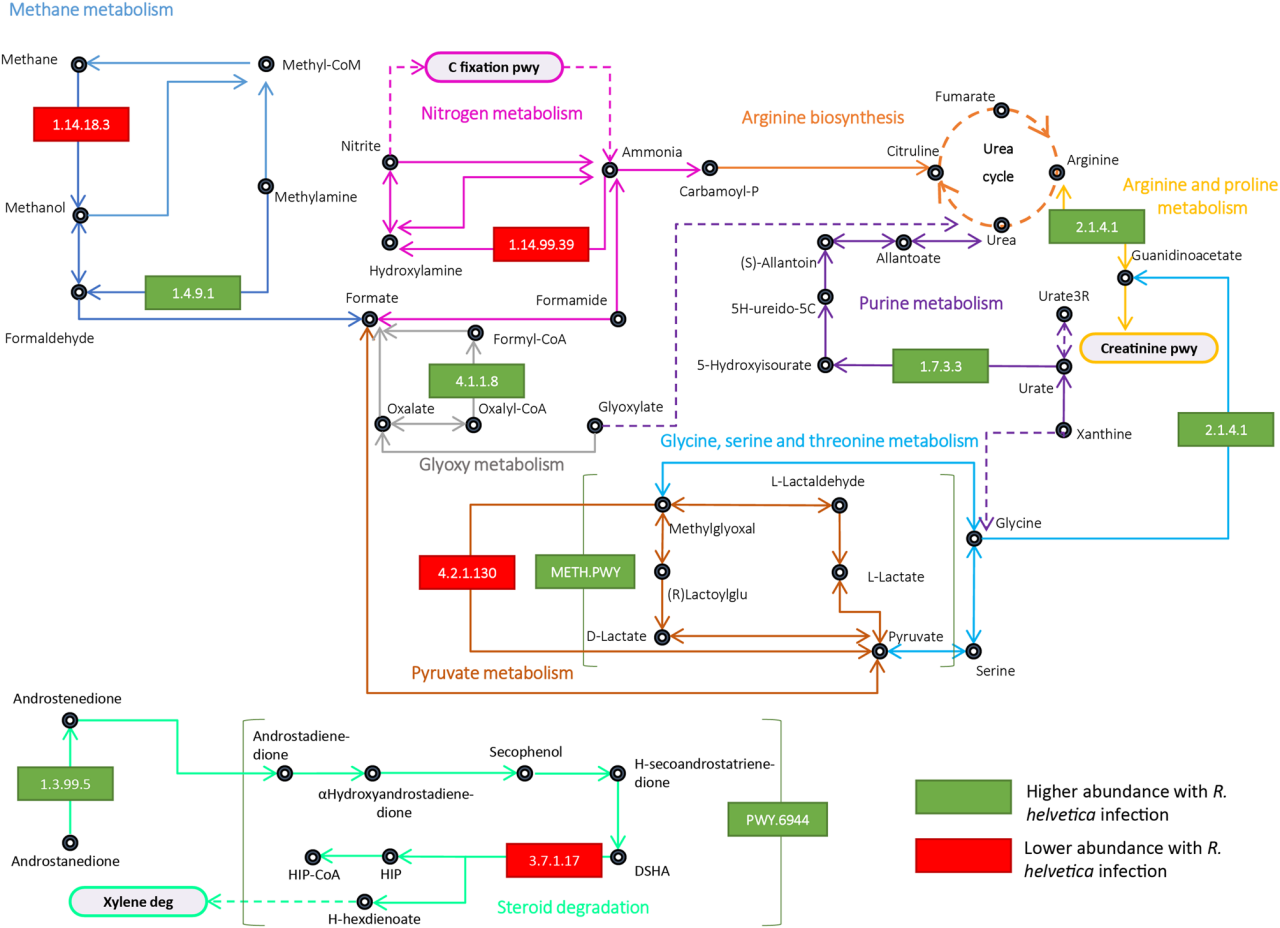
Partial reconstruction of microbial metabolic pathways in the tick microbiome. Partial reconstruction of methane, nitrogen, arginine and proline, glycine, serine and threonine, glyoxylate and dicarboxylate, purine, pyruvate, glycan biosynthesis and steroid degradation metabolic pathways is displayed. Only metabolic enzymes and pathways with significant differences in relative abundances (*p* < 0.05) between *R. helvetica-*positive and pathogen-free *I. ricinus* ticks are represented. KEGG annotation were included in this analysis. Continuous lines represent direct reactions catalyzed by enzymes. Dashed lines represent reconstruct reactions with shortcuts. Oval boxes represent pathway reconstruction based in KEGG. Enzymes with significant abundance difference are represented in a green rectangle, if they have a higher abundance, and in red, if they have a lower abundance, in *R. helvetica*-positive compared to pathogen-free samples. Pathway with a significant difference of abundance between pathogen-free and *R. helvetica-*positive samples are represented in brackets, in green or in red if the abundance is higher or lower with *R. helvetica* presence. C fixation pwy, Carbon fixation pathways in prokaryotes; Carbamoyl-P, Carbamoyl phosphate; Meth.pwy, Methgyut.pwy; Creatinine pwy, Creatinine pathway; Glyoxy meth, Glyoxylate and dicarboxylate metabolism; Xylene deg, Xylene degradation; G8 Glycane G00008; G9, Glycane G00009; D-diphospate, Dolichyl diphosphate; Protein asp, Protein asparagine; Androstadienedione, Androsta-1,4-diene-3,17-dione; αHydroxyandrostadienedione, 9 alpha-Hydroxyandrosta-1,4-diene-3,17-dione; H-secoandrostatrienedione, 3,4-Hydroxy-9,10-secoandrosta-1,3,5(10)-triene-9,17-dione; H-hexdienoate, 2-Hydroxy-cis-hex-2,4-dienoate; (R)-Lactoylglu, (R)-S-Lactoyl-glutathione; Urate3R, Urate-3-ribonucleoside; 5H-ureido-5C, 5-Hydroxy-2-oxo-4-ureido-2,5-dihydro-1H-imidazole-5-carboxylate.

We checked if the changes observed in the composition and the structure of the microbial communities had an impact on the inferred functional profile of tick microbiome. We analyzed and compared the composition, diversity and relative abundance of predicted enzymes and metabolic pathways in the microbiome of the pathogen-free and the *R. helvetica-*positive ticks. The two datasets shared 2175 (94.9%) of the 2293 total of predicted enzymes (Fig. 5A). The pathogen-free and *R. helvetica-*positive ticks also had 412 (97.4%) of the 423 total functional pathways in common (Fig. 5B). Bray–Curtis index and a PERMANOVA test demonstrated that the beta-diversity of enzymes (Fig. 5C) and pathways (Fig. 5D) was not significantly different between the two datasets. However, an ANOVA test demonstrated that the number of predicted enzymes of pathogen-free samples were more variable than the *R. helvetica* infected one (Fig. 5C). Differential analysis showed that the relative abundance of predicted genes (KO) encoding 13 enzymes (Fig. 5E) and 2 pathways (Fig. 5F) changed significantly between the two datasets. These 13 enzymes were involved in various metabolic functions including energy metabolism (methane and nitrogen metabolism), amino acid metabolism (arginine and proline metabolism as well as glycine, serine and threonine metabolism), glycan metabolism (glycan biosynthesis), nucleotide metabolism (purine metabolism), pyruvate metabolism, carbohydrate metabolism (glyoxylate and dicarboxylate metabolism) and steroid degradation (Fig. 6). In the methane metabolism pathways, the methane monooxygenase (EC:1.14.18.3) and methylamine dehydrogenase (EC:1.4.9.1) had a significantly lower and higher abundance, respectively, in the *R. helvetica-*positive ticks compared with the pathogen-free ticks. The methane monooxygenase is the first enzyme in the metabolic pathway of methanotrophs bacteria^29^. For those bacteria, methane is the sole source of carbon and energy. In pyruvate metabolism, the D-lactate dehydratase enzyme (EC:4.2.1.130) had a lower abundance, and the super pathway of methylglyoxal degradation (for transformation of methylglyoxal in pyruvate) had a higher abundance in *R. helvetica-*positive tickscompared with pathogen-free ticks. Methylglyoxal is produced in small amounts via several metabolic pathways and is highly toxic in various animal species^30^.

The predicted gene encoding for the enzyme ammonia monooxygenase (EC:1.14.99.39), which participates in the nitrogen metabolism, had a lower abundance in *R. helvetica*-infected samples. In contrast, the 3-oxo-5alpha-steroid 4-dehydrogenase (EC:1.3.99.5), which is an enzyme catalyzing one of the initial steps in the steroid degradation pathway, had higher abundance in *R. helvetica-*positive ticks. This was also the case for the androstenedione degradation pathway (PWY.6944). Androstenedione is a key intermediate of microbial steroid metabolism^31^. The enzyme glycine amidinotransferase (EC:2.1.4.1), an enzyme in the arginine and proline metabolic pathway as well as in glycine, serine and threonine metabolism, was more abundant in *R. helvetica-*positive ticks. The glucose-6-phosphate dehydrogenase (EC:1.1.98.2), a key enzyme of the classical Entner–Doudoroff (ED) pathway in bacteria, catalyzing the oxidation of glucose-6-phosphate to gluconolactone-6-phosphate with either NADP + or NAD + as electron acceptors^32^, was more abundant in *R. helvetica-*positive ticks (Fig. 5C). The mercury (II) reductase (EC:1.16.1.1) enzyme serves to convert toxic mercury into inert elemental mercury^33^, this enzyme was less abundant in *R. helvetica-*positive ticks (Fig. 5C).

## Discussion

Evidence from field^26^ and laboratory^34^ studies suggest that tick colonization by obligate intracellular *Rickettsia* modulates the tick microbiota. The presence of *Rickettsia* in questing *I. ricinus* nymphs was associated with changes in the abundance of bacterial taxa compared to *Rickettsia*-negative samples^26^. Similar to the effect of *R. helvetica* infection on *Ixodes* microbiota observed in this study (i.e., reduction in the bacterial diversity), *A. phagocytophilum* infection also induced a significant reduction of the bacterial diversity in infected ticks^28^. In contrast, field-collected *Borrelia*-positive adult female *I. scapularis* had significantly greater bacterial diversity than *Borrelia*-negative ticks^35^, which is consistent with the finding by Narasimhan et al.^27^ that laboratory-reared *I. scapularis* ticks with greater microbiome diversity were more successfully colonized by *B. burgdorferi*. Also, *I. ricinus* nymphs that had taken their larval blood meal from an *Borrelia afzelii*-infected mouse had a more diverse bacterial microbiome than the control nymphs^36^. Comparing the effect of intracellular (i.e., *A. phagocytophilum* and *R. helvetica*) and extracellular (i.e., *Borrelia*) bacteria on the tick microbiota one could deduce a pattern in which intracellular pathogens decrease, while extracellular pathogens increase the tick microbiota diversity. However, other studies found that field-collected *Borrelia*-infected *Ixodes pacificus* nymphs had lower species evenness than uninfected ticks^37^, while no association between the diversity of the *I. scapularis* microbiome and its probability of carrying *B. burgdorferi* was found in another study using ticks from a region with a high incidence of Lyme disease^38^. This variability in results encourage further comparative studies on the impact of intracellular and extracellular pathogen infection on the tick microbiota under laboratory conditions and in ticks collected in the field.

*Rickettsia* infection has also been associated with changes in the abundance of several bacterial taxa in the tick microbiome. Particularly, *Ca.* Midichloria, *Pseudomonas*, *Bacillus* and Rhizobiaceae were significantly more abundant in *Rickettsia*-positive samples while *Spiroplasma* was significantly less abundant^26^. Similarly, in this study we found higher abundance of Ca. *Midichloria* in *R. helvetica*-positive compared to pathogen-free samples. In the work by Lejal et al.^26^, network analysis revealed that *I. ricinus* nymphs positive for *Rickettsia* had 17 significant partial correlations, five of them were negative. Several members of the *Rickettsia* genus, notably *R. helvetica*, were positively correlated to Ca. *Midichloria* and *Pseudomonas*, and one *Rickettsia* OTU was negatively correlated to *Bacillus*^26^. Interestingly, in our study we also found *Bacillus* to be negatively correlated with the taxon *Rickettsia* in the network of *R. helvetica*-positive samples. Altogether these results suggest that *R. helvetica* and *Bacillus* might compete and that Ca. *Midichloria* may enhance *R. helvetica* growth within *I. ricinus*. In this study, the abundance of Rhizobiaceae was not significantly affected by the presence of *R. helvetica* and Rhizobiaceae was not directly linked to *Rickettsia* in the network of *R. helvetica*-positive sample. However, Rhizobiaceae was positively correlated with the keystone taxon *Sphingopyxis* (Fig. 4B). These results suggest an indirect interaction between *Rickettsia* and Rhizobiaceae via the keystone taxon *Sphingopyxis*. *Rickettsia helvetica* infection could induce changes in the microbial community that favors a predominant role of *Sphingopyxis*, which in turn favors Rhizobiaceae colonization. Despite ruling out the presence of 25 bacterial species (including 6 *Rickettsia* species), and 7 parasite species in the ‘pathogen-free’ samples by microfluidic PCR; and of those tick-borne pathogens, the *R. helvetica*-positive samples were positive only in *R. helvetica* infected ticks (see “Materials and methods”), it is important to mention that the 16S rRNA amplicon sequence analysis revealed the presence of the taxon *Rickettsia* in 73% of the pathogen-free samples. This suggests the possibility that undescribed *Rickettsia* pathogens, or perhaps *Rickettsia* endosymbionts, which are frequently found in *Ixodes* ticks^14^, may be present in the pathogen-free and *R. helvetica*-positive samples. Although the possible presence of co-infections^39^ and/or endosymbionts^14^ may affect the tick microbiome, and therefore the interpretation of the results of the present study, working with gnotobiotic ticks (with a defined microbiota composition) is currently beyond the state of tick research.

The mechanisms by which *R. helvetica* modulates the taxonomic and functional profiles of the tick microbiome are yet to be determined. Similar to our results with *R. helvetica*, modulation of taxonomical profiles by *A. phagocytophilum* infection was associated with changes in the relative abundance (fold changes) of several genes (KO) coding for enzymes in the *I. scapularis* microbiome, as predicted with Phylogenetic Investigation of Communities by Reconstruction of Unobserved States 2 (PICRUSt2)^40^. The infection with *A. phagocytophilum* changed the relative importance (measured as changes in weighted degree) of several pathways in response to pathogen infection^40^. Notably, the microbiome disturbed by *A. phagocytophilum* had a decreased importance of PICRUSt2-predicted polysaccharide biosynthesis and degradation pathways^40^, which was consistent with the experimental determination of the decrease in exopolysaccharide poly-*N*-acetylglucosamine (PNAG, a major component of bacterial biofilms) in tick midgut infected with *A. phagocytophilum*^28^. The decrease in biofilms in the midguts of *A. phagocytophilum*-infected ticks was caused by the binding of IAFGP to the terminal d-alanine residue of the pentapeptide chain of bacterial peptidoglycan which altered permeability and decreased the capacity of Gram-positive bacteria to form biofilms^28^. *Anaplasma phagocytophilum* induces ticks to express IAFGP, an antimicrobial protein with the ability to alter microbiota composition^28^ and was associated with the formation of scattered and diffused bacterial biofilms^41^. Interestingly, the tick gene *pixr*, upregulated by the extracellular bacteria *B. burdorferi*, encodes the protein PIXR which also inhibits Gram-positive bacterial biofilm formation by a mechanism that remains to be determined^27^. Larval ticks that fed on PIXR-immunized mice demonstrated differences in the microbiome composition when compared to larvae that fed on control (ovalbumin-immunized) mice. This suggests that in the absence of PIXR, gram-positive bacterial biofilms likely increase and consequently shift the microbiome composition^27^. Furthermore, the microbiome of larval ticks fed on PIXR-immunized mice was associated with an increase in the representation and importance of PICRUSt2-predicted polysaccharide biosynthesis pathways^40^. Thus, tick gene expression modulation by *A. phagocytophilum* and *B. burdorferi* may explain the impact of tick-borne pathogens on Gram-positive bacteria of the tick microbiota and the capacity of these bacteria to form biofilms. A similar mechanism could explain potential competitive interactions between the Gram-positive bacterium *Bacillus* and the intracellular pathogen *R. helvetica*. Further research is needed to examine whether *R. helvetica* infection modulates the tick transcriptional response to induce the expression of proteins with microbiota-modulating effects.

Differences in the hierarchical organization of *R. helvetica*-positive and pathogen-free samples suggest that the microbial ecology within *I. ricinus* changes in response to pathogen infection. Hierarchical re-organization of tick microbial communities was previously reported in *I. scapularis* ticks under heat stress^42^, and *I. ricinus* exposed to anti-microbiota vaccines^43^. Notably, heat stress was associated with significant changes in the taxonomical profiles^44^ and microbial community re-structuration of *I. scapularis* microbiota, but a core of four taxa (i.e., *Pseudomonas*, *Ralstonia*, *Acinetobacter* and *Bradyrhizobium*) were still identified as keystone regardless of the differences in temperature (i.e., 4, 20, 30, and 37 °C) at which ticks were incubated^42,44^. The results suggest that the structure of tick microbial communities is more sensitive to biological disturbance by pathogen infection than to environmental stressors such as temperatures of 37 °C, which can be considered high for the ticks *I. scapularis* and *I. ricinus*. In agreement with this idea, previous studies have shown that *A. phagocytophilum* infection reshapes the co-occurrence of bacteria in microbial communities in the tick guts, reducing dramatically the connections between modules, reflected in the reduction in the average clustering coefficient from 0.528 in uninfected ticks to 0.376 in *A. phagocytophilum*-infected ticks^40^. A similar effect was observed for *R. helvetica* as the pathogen-positive network had reduced average clustering coefficient from 0.644 to 0.525, and modularity from 1.368 to 0.836, in comparison with uninfected ticks.

## Conclusions

In this study, we showed that the presence of *R. helvetica* is associated with microbiome modulation in *I. ricinus* collected from humans. *Rickettsia helvetica* is an emerging rickettsial species isolated from ticks in various European countries^45^ such as Switzerland^46^, Germany^47^, France^48^, Hungary^49^, and The Netherlands^50,51^. This bacterium was also recently identified in ticks collected from humans in Serbia^4,52^, and it has been associated with clinical manifestations in human patients^53^. Others have reported that *R. helvetica* infection may be an indirect cause in cases of acute perimyocarditis^54^, and unexplained febrile illness^55,56^. The characterization of the tick microbiome in ticks collected from humans can reveal novel targets to prevent tick-borne pathogen infection, as the tick microbiome has a critical role on vector competence. Recent research has focused on vaccination targeting vector microbiota to alter tick microbiota^43,57^, reduce vector competence and block pathogen transmission^58^. From the results in this study, we hypothesize that an anti-microbiota vaccine targeting the tick endosymbiont *Ca.* Midichloria may affect the performace of *R. helvetica* within ticks with implications for the acquisition and/or transmission of *R. helvetica* to humans.

## Materials and methods

### Study design

The study aimed to characterize the impact of *R. helvetica* infection on the microbiome of *I. ricinus* ticks collected from humans. For this, we used DNA samples obtained from ticks (at the nymph stage and with a feeding period < 72 h) collected from humans in a previous study^4^. The presence of tick-borne pathogens in *I. ricinus* ticks was assessed using high-throughput microfluidic real-time PCR (Fluidigm, San Francisco, CA, USA) as described^4^. In the previous study^4^, the ticks were morphologically identified as *I. ricinus* and tested for the presence of 25 bacterial pathogens (*B. burgdorferi *sensu stricto*, Borrelia garinii, B. afzelii, Borrelia valaisiana, Borrelia lusitaniae, Borrelia spielmanii, Borrelia bissettiae, Borrelia miyamotoi, Anaplasma marginale, Anaplasma platys, Anaplasma phagocytophilum, Anaplasma ovis, Anaplasma centrale, Anaplasma bovis, Ehrlichia canis, Neoehrlichia mikurensis, Bartonella henselae, Francisella tularensis, Coxiella burnetii, Rickettsia conorii, Rickettsia slovaca, Rickettsia massiliae, Rickettsia aeschlimannii, Rickettsia felis* and *Rickettsia helvetica*), and 7 parasite species (*Babesia microti, Babesia canis, Babesia ovis, Babesia bovis, Babesia caballi, Babesia venatorum,* and *Babesia divergens*). For this study, selected samples were classified in two categories: (i) ‘pathogen-free’ samples (n = 11) that were negative for the above tick-borne pathogens, and (ii) ‘*R. helvetica*-positive samples (n = 18) that were negative for the listed pathogens except *R. helvetica*. Infection by *R. helvetica* in these samples was confirmed by conventional nested PCR^4^.

Overall, high-throughput amplicon sequencing of 16S rRNA gene was used to compare the microbiobial community composition, diversity indexes (i.e., alpha-diversity and beta diversity) and to detect the differential abundant taxa between pathogen-free and *R. helvetica*-positive samples. We used the ‘Sparce Correlations for Compositional data’ (SparCC^59^) method to infer microbial co-occurences networks and compare their topological features. We further studied the interaction patterns of the microbial communities by identifying keystone taxa^60^. The functional traits of the tick microbiome were inferred using PICRUSt2^6^ and explored for possible association with *R. helvetica* infection.

### DNA extraction and 16S rRNA sequencing

Genomic DNA was extracted and purified as described previously^4^. More than 900 ng of DNA at ≥ 20 ng/μL concentration was amplicon sequenced for the bacterial 16S rRNA gene by Novogene Bioinformatics Technology Co. (London, UK). Libraries were prepared with NEBNext® Ultra™ IIDNA Library Prep Kit (New England Biolabs, MA, USA). A single lane of Illumina MiSeq system was used to generate 251-base paired-end reads from the V4 variable region of the 16S rRNA gene using bar-coded universal primers (515F/806R) in ticks. Two extraction reagent controls were included, in which the different DNA extraction steps were performed using the same conditions as for the samples but with water as the template. DNA amplification was then performed on the extraction controls under the same conditions as the other samples. The raw 16S rRNA sequences obtained from tick samples were deposited at the SRA repository (Bioproject No. PRJNA803003).

### Processing of raw sequences

The analysis of 16S rRNA sequences was performed using QIIME2 pipeline (v.2019.7)^62^. The raw sequences (demultiplexed in fatsq files), were denoised, quality trimmed and merged using the DADA2 software^63^ implemented in QIIME2^62^. The ASVs were aligned with q2-alignment of MAFFT^64^ and used to construct a phylogeny with q2-phylogeny of FastTree 2^65^. Taxonomy was assigned to ASVs using a classify-sklearn naïve Bayes taxonomic classifier^66^ based on SILVA database (release 138)^67^. Only the target sequence fragments were used in the classifier (i.e., classifier trained with the 515F/806R primers)^68,69^. Taxa that persisted across serial fractions of the samples using QIIME 2 plugin feature-table (core-features) were considered ubiquitous^62^.

### Diversity indexes and taxa abundance

The alpha diversity provides a summary of the microbial community in individual samples on the richness (number of species) and evenness (how well each species is represented). Alpha diversity metric was estimated using the q2-diversity plugin in Qiime2 environment. The alpha diversity richness was explored using observed features^70^, and the evenness with the Pielou evenness index^71^. Differences in alpha-diversity metric between groups were assessed using Kruskal–Wallis test (alpha = 0.05) using QIIME 2^62^ and GraphPad Prism version 8.0.1 (GraphPad Software, San Diego, California USA). Beta diversity is a measure of diversity between individual samples that assesses similarity of communities compared with the other samples analyzed. Bacterial beta-diversity was assessed using the Bray Curtis dissimilarity index^72^, and compared between groups using the PERMANOVA test (*p* < 0.05) realized with QIIME 2^62^ and Past 4 version 4.08 (2001). Beta dispersion was calculated using betadisper function and Vegan script implemented in Rstudio^73^. Beta dispersion statistical analyses were performed using ANOVA test (*p* < 0.05).

The taxa abundance differences were tested using a *t*-test with a realized with ANOVA-Like Differential Expression (ALDEx2) package^74^ in R-studio^73^. Microbial diversity analyses were carried out on rarefied ASV tables, calculated using the q2-diversity plugins. Only taxa with a significant uncorrected *p*-value (*p* < 0.05) were used for representation of the taxa abundance. Relative abundance was measured as centered log ratio (clr), calculated with ALDEx2 package^74^ implemented in R-studio^73^. The resulting data were used to construct the heatmap with the heatmap.2 function implemented in R-studio environment^73^.

### Inference of bacterial co-occurrence networks and statistical network estimation

Co-occurrence networks were built for the two sets of samples using the taxonomic profiles at genera level. The networks allow the graphic visualization of the microbial community assemblies. Bacterial taxa are represented by nodes and the significant correlations between taxa are represented by edges. Analyses of significant positive (weight > 0.75) or negative (weight < − 0.75) correlations were performed using the SparCC method^59^ implemented in R studio environment^73^. The visualization and assessment of the networks were performed using the software Gephi 0.9.2^75^. Network topological features were calculated: number of nodes and edges, network diameter (the shortest path between the two most separated nodes), modularity (the strength of the division of a network into modules), average degree (the average number of links per nodes), weighted degree (the sum of the weight of all the edges connected to a node) and clustering coefficient (the degree to which nodes in a network tend to form clusters).

### Differential network analysis

To compare the networks of pathogen-free and *R. helvetica*-positive *I. ricinus* ticks, a statistical network estimation analysis was conducted with network construction and comparison for microbiome (NetCoMi) method^76^ implemented in R studio^73^. To test for dissimilarities between the two networks, Jaccard index was calculated for degree, betweenness centrality, closeness centrality and eigenvector centrality. The Jaccard index tests the similarity between sets of “most central nodes” of networks, which are defined as those nodes with a centrality value above the empirical 75% quartile. Thus, this index expresses the similarity of the sets of most central nodes as well as the sets of hub taxa between the two networks. Jaccard index range from 0 (completely different sets) to 1 (sets equal). The two *p*-values P (J ≤ j) and P (J ≥ j) for each Jaccard’s index are the probability that the observed value of Jaccard’s index is ‘less than or equal’ or ‘higher than or equal’, respectively, to the Jaccard value expected at random which is calculated taking into account the present total number of taxa in both sets^77^. The ARI was calculated to test the dissimilarity of clustering in the networks. ARI values range from − 1 to 1. Negative and positive ARI values mean lower and higher than random clustering, respectively. An ARI value of 1 corresponds to identical clustering, and 0 to dissimilar clustering. The *p*-value test if the calculated value is significantly different from zero^76^.

### Keystone taxa identification

Keystone taxa were defined by three criteria as previously reported^43,57^: (i) ubiquitousness (microbial taxa present across all the samples of an experimental group), (ii) eigenvector centrality higher than 0.75, and (iii) high mean relative abundance (i.e., higher than that of the mean relative abundance of all taxa in an experimental group). The eigenvector centrality measures the influence of a node in a network, a high eigenvector score means that a node is connected to many nodes which themselves have high scores^78^. Eigenvector centrality values were exported from Gephi 0.9.2 software^75^. For each sample in both data sets, the mean clr value was calculated and plotted together with eigenvector centrality values using GraphPad Prism version 8.0.0 (GraphPad Software, San Diego, California USA).

### Prediction of functional traits in the tick microbiome

The 16S rRNA amplicon sequences from each data set were used to predict the metabolic profiling of each sample. PICRUSt2^61^ implemented in QIIME2 was used to predict the metagenomes from 16S rRNA amplicon sequences. The ASVs were placed into a reference tree (NSTI cut-off value of 3) containing more than 20,000 full 16S rRNA sequences from prokaryotic genomes, which is then used to predict individual gene family copy numbers for each ASV. The predictions are based on Kyoto Encyclopedia of Genes and Genomes (KEGG) orthologs (KO)^79^. The output of these analyses included pathways and EC (Enzyme Commission number) profiling. The pathways were constructed based on the MetaCyc database^80^.

### Ethics statement

Ticks from tick-infested patients were collected in the context of a previous study^4^, under a protocol approved by the ethical committee of Pasteur Institute Novi Sad (Ethical approval No. 03/2019) and conducted according to Declaration of Helsinki and The Patient Rights Law of the Republic of Serbia. Oral and written informed consents were obtained from the adult individuals, or from the parents or legal guardians of the children.

## Data Availability

The data that support this study results are available on the SRA repository (Bioproject No. PRJNA803003).
